# Screening for balance in children and adults in a community science education setting: Normative data,  influence of age, sex, and body mass index, and feasibility

**DOI:** 10.1371/journal.pone.0268030

**Published:** 2022-05-18

**Authors:** Phyllis Friello, Nathan Silver, Haleh Sangi-Haghpeykar, Helen S. Cohen

**Affiliations:** 1 Applied Research Collaborative, Space Center Houston, Houston, TX, United States of America; 2 Department of Otolaryngology–Head and Neck Surgery, Baylor College of Medicine, Houston, TX, United States of America; 3 Department of Obstetrics and Gynecology, Baylor College of Medicine, Houston, TX, United States of America; University of Rochester, UNITED STATES

## Abstract

**Background:**

Screening standing and walking balance is useful for people suspected of having vestibular disorders, a variety of neurologic and musculoskeletal disorders, and for screening astronauts returning after exposure to microgravity. Visitors to a community science education center children and adults, aged 4 to 85, were tested on tandem walking with eyes closed and the modified Romberg test on compliant foam. They were then asked about their experience participating in research, many people for the first time.

**Methods:**

Subjects performed 10 steps of tandem walking with eyes closed, and three trials of the modified Romberg, or Clinical Test of Sensory Integration and Balance, with eyes closed, standing on compliant memory foam, with a) head still, b) head shaking in yaw, and c) head nodding in pitch. Afterward, staff queried subjects about the experience of participating in science.

**Results:**

Age-related changes across the life span occurred in both sets of tests. Therefore, look-up tables by age are provided. Body mass index significantly affected tandem walking. Some sex differences were found. The tests were easy to administer in a community setting. Most participants enjoyed the experience and reported that they learned about the process of scientific research.

**Discussion:**

These data support and extend the evidence for age-related changes in balance performance across the lifespan and for an influence of body mass index on some balance skills. Clinicians and sports educators should be cognizant of these differences when they use these tests for screening. The community science education environment provided a useful laboratory in which to collect valid and reliable data, while simultaneously educating participants about the process of science.

## Introduction

Balance impairments are hallmarks of vestibular disorders [1, 2]. They are also characteristic of astronauts during the acute stage of recovery from exposure to microgravity [3–5]. As a result, tests of balance are useful for screening diverse groups of people. The equipment which is often used to test people for vestibularly-mediated balance problems, such as computerized dynamic posturography [1], is too large to transport to health clinics, sports stadiums and other community events, and landing sites for returning crew members and may be too costly for some clinical, educational and athletic facilities. Therefore, simple screening tests have been developed, such as the modified Clinical Test of Sensory Integration and Balance (CTSIB), based on the old Romberg test [6] but updated with continuously compliant memory foam [7, 8] and sharpened but simplified with 3 conditions, two of which involve augmented head motions [9–11]. Ten steps of tandem walking with eyes closed (TW) is also useful for screening adult patients and returning crew members [12–14]. These tests may also be useful for testing children and adults who incur mild head trauma during participation in sports that causes balance problems. The simplicity and portability of these tests might make them particularly useful for remote areas where sophisticated diagnostic testing is unavailable and rapid screening is desirable.

Normative data on children have not been published for either CTSIB or TW. Some evidence suggests that weight affects balance [15–19] but only one published study has examined weight and balance testing on foam [20]. Body mass index, i.e. weight relative to height, is a better independent measure than weight, alone. To study these questions a larger sample was needed than was readily available in the senior investigator’s laboratory setting.

In a happy convergence of interests, data for this study were collected at Space Center Houston (SCH). SCH is a private, science education center focused on space exploration. It received 1.9 million visitors per year prior to the COVID-19 pandemic. SCH staff frequently inform visitors about research related to crew health. The senior investigator is a clinician/ scientist who has collaborated with NASA investigators and who could explain how data from this study might be used by space physiologists and flight surgeons to screen astronauts after long duration space flight and also to screen people in the community with balance impairments.

The scientific goals were: 1) to develop benchmark data on children, 2) to determine if children differed from adults in their responses on these common screening tests, 3) to determine the effects of body mass index and demographic factors on test performance, and 4) to develop handy look-up tables for use by clinicians. The educational goals were 1) to determine if visitors in a community setting learned about the process of research by participating in it and 2) if they felt positive about the experience. The goals of the study were met.

## Materials and methods

### Subjects

Participants were 1869 visitors to Space Center Houston, aged 4 to 85 years (mean age 24.8 years, SD = 17.2; 868 (46.5%) male, 998 (53.5%) female, 3 unknown sex), between June 2019 and September 2021. A table was set up in the testing area with signs inviting visitors to participate. Many visitors inquired what the staff were doing. The staff also spoke with the parents of children who arrived for Space Camp activities. See Table 1 for demographic details. Questionnaires were used to screen out potential subjects with histories of neurologic, musculoskeletal, and otologic disorders; including treatment for dizziness, vertigo or imbalance.

**Table 1:** Descriptive data about the sample. The total sample size was 1869.

Characteristic	No. (%) of participants
Age (Years)	
4+5	76 (4.07)
6+7	158 (8.45
8+9	184 (9.84)
10+11	177 (9.47)
12+13	135 (7.22)
14+15	85 (4.55)
16+17	61 (3.26)
18+19	66 (3.53)
20–25	136 (7.28)
25–30	187 (10.01)
30–35	124 (6.63)
35–40	104 (5.56)
40–45	104 (5.56)
45–50	72 (3.85)
50–55	63 (3.37)
55–60	50 (2.68)
60–65	35 (1.87)
65–70	27 (1.44)
70–75	13 (0.70)
75–80	8 (0.43)
80–85	4 (0.21)

Written informed consent was obtained prior to participation for all subjects. Adults gave written informed consent, themselves. Children gave assent to the extent that they were able and their parents gave written informed consent. This study was approved by the Institutional Review Board for Human Subjects Research for Baylor College of Medicine and Affiliated Hospitals.

After giving written informed consent, subjects were asked to complete a form to describe their age, sex, and height and weight in order to calculate body mass index (BMI). The categories of overweight and obesity were defined for adults using the definitions by the Centers for Disease Control and Prevention [21]: overweight meant body mass index 25.0 to < 30, and obesity meant body mass index ≥ 30. For children the definitions by the World Health Organization [22] were used: overweight meant > 1 SD for age norms and obesity meant > 2 SD for age norms.

### Materials and training

Data were collected by 15 members of the education staff. The staff members were full- and part-time instructors experienced in interactions with all age groups. Prior to their participation the staff were all trained in Human Subjects Research Protection using training materials from the National Institutes of Health web page. Prior to the onset of the COVID-19 pandemic the staff were trained to administer the tests in person. Immediately after the onset of the pandemic, SCH was closed to visitors. After visits to SCH resumed on a limited basis with COVID-19 protocols in place, the staff met with one of the investigators via Zoom to review the testing procedures.

No materials were needed for TW. For CTSIB a handheld stopwatch was used to measure the duration of each trial. Subjects stood on slab of medium density memory foam (Sunmate foam, PSI = 93, 71 X 62 X 10 cm, with a 2 mm polyurethane (Skinsoft) coating on one side (Dynamic Systems, Inc, Leicester, NC,; sunmatecushions.com). A digital metronome was used to indicate the 0.3 Hz frequency needed.

### Procedures

#### Scientific methods

Testing was performed in a semi-secluded alcove of the main building at SCH, using previously established protocols [11, 13]. To standardize footwear and to maintain hygiene, subjects were tested without shoes but while wearing socks. They were allowed to rest in between trials as needed. All subjects were given the same instructions.

For TW, subjects initially performed a practice trial of 5 steps, heel-to-toe, with eyes open and arms crossed at the waist, to be sure that they understood the nature of the task. Then, they performed a single experimental trial of 10 heel-to-toe steps with eyes closed and arms crossed at the waist. Errors were: opening the eyes, moving the arms, taking a side step, leaving more than 2 cm between the front and rear feet. The dependent measure was the total number of correct steps out of 10; correct steps did not have to be consecutive.

For all three CTSIB trials subjects stood on the foam with feet adjacent to each other, arms crossed at the waist, head upright. They had 5 to 10 seconds of practice with eyes open and then performed the three experimental trials with eyes closed for up to 30 sec per trial: 1) head stationary, i.e. not deliberately moving, or head still; 2) head shaking leftward-rightward as if indicating “No” at 0.3 Hz, or head yaw, and 3) head nodding upward-downward as if indicating “Yes” at 0.3 Hz, or head pitch. Prior to the head yaw and pitch trials, to learn the correct frequency each subject practiced the head movements while standing on the floor with eyes open. Then, the test trials were performed on foam with eyes closed. The dependent measure was the duration of the trial before an error, up to 30 sec. Errors were opening the eyes, moving the arms or feet, or leaning backward against the wall.

#### Educational methods

Because this study was performed at an education center, and data were collected by educators, we wanted to determine if the study contributed to the educational goals of the facility. Therefore, after they performed the study tests, as described in the scientific methods, participants were asked a series of questions about their experience. We asked if they enjoyed it and we asked what they learned. We also wanted to know if this novel experience of data collection was interesting for the education staff without being onerous, and if they thought it had educational value based on their objective observations. Therefore, the education staff were asked for their opinions, too.

#### Statistical methods

General linear regression was used to assess the association between CTSIB and age, where least square means, and 95% confidence intervals (CI) are presented for various age categories. For TW (a non-normally distributed variable), we present median and interquartile range (25-75^th^ percentile) by age. For all other comparisons, Wilcoxon rank sum tests or Kruskal Wallis tests were used as needed when comparing two or more than two groups on different parameters of interest. Analyses were performed in SAS statistical software (version 9.4 Cary, NC). Statistical significance was set at p<0.05.

## Results

### Scientific results

**Fig 1:**
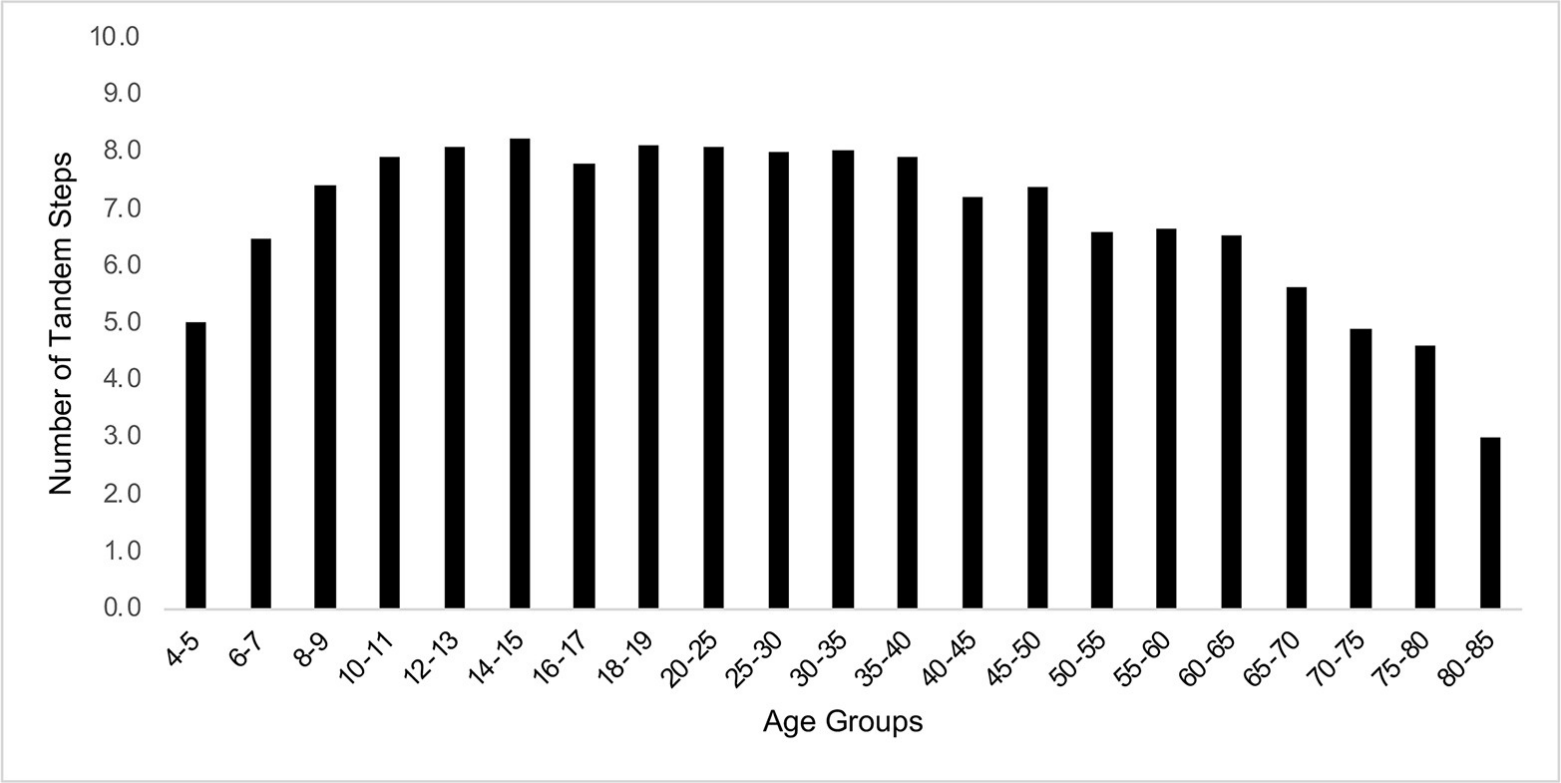
Median TW data by age groups, with sex, BMI and race/ ethnicity collapsed. The data are the number of correct tandem steps.

**Fig 2:**
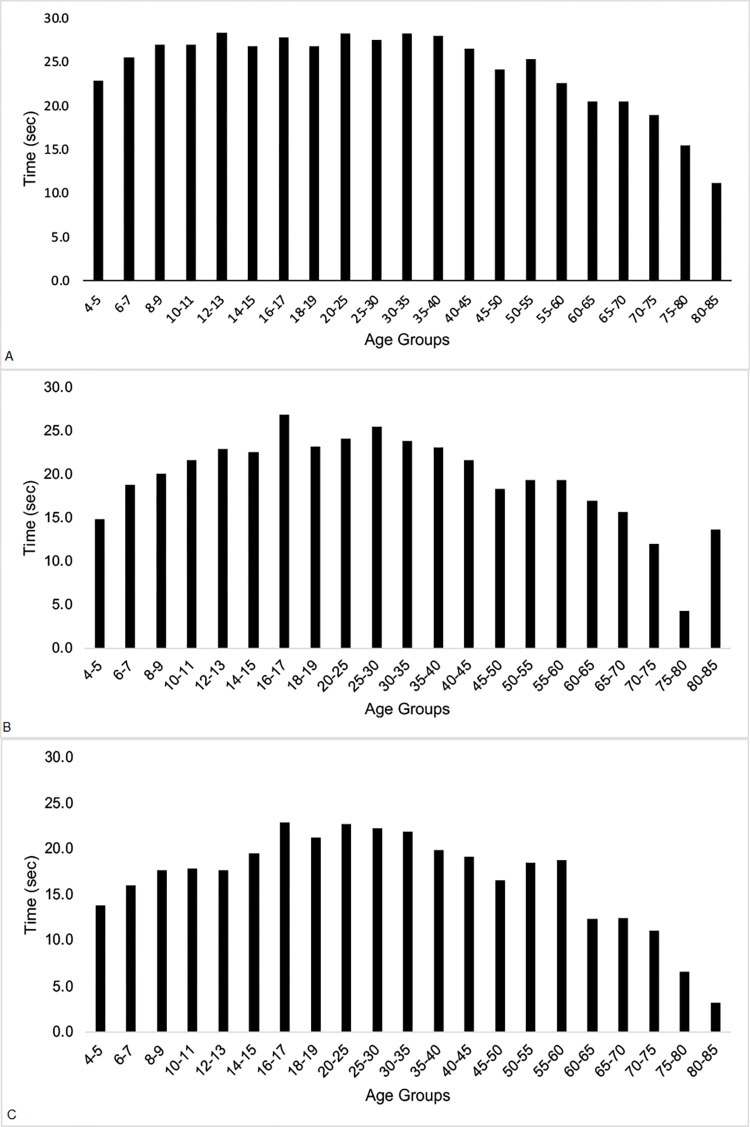
A-C. Mean CTSIB data by age groups, with sex, BMI and race/ ethnicity collapsed. A, CTSIB head still; B, CTSIB head yaw; C, CTSIB head pitch.

Age-related changes were found in trial duration in all three CTSIB conditions and in number of correct tandem steps in TW, as shown in Figs 1 and 2. In general, for TW, subjects aged 4 to 9 and aged ≥ 60 had fewer correct tandem steps than subjects aged 10 to 59 years. In general, for CTSIB, head still, head yaw and head pitch, subjects aged 4 to 7 and ≥ 60 performed the test for less time than older children and young to middle-aged adults. The details varied by test condition, however. Data from the two oldest groups of subjects should be considered as preliminary due to the small sample sizes.

Tables 2 and 3 show measures of central tendency and ranges of data for TW and CTSIB, respectively, for potential use by clinicians. Some age groups differed significantly. Because some noncontiguous age groups differed significantly but some contiguous age groups did not differ, no simple graphical method to show those differences was possible. More importantly, these data can be used by clinicians—in health care, education, sports and other professions that might use these tests–as handy look-up tables for easy reference.

**Table 2:** TW by age, showing median and interquartile range. Use the data from upper ages with caution due to small sample sizes.

Age range (years)	Median (Interquartile range)
4.0 to 5.9	5 (2 to 8)
6.0 to 7.9	7 (5 to 9)
8.0 to 9.9	8 (6 to 9)
10.0 10 11.9	8 (7 to 10)
12 to 13.9	9 (7 to 10)
14 to 15.9	9 (8 to 10)
16 to 17.9	8 (7 to 10)
18 to 19.9	9 (7 to 10)
20 to 24.9	9 (7 to 10)
25.0 to 29.9	9 (7 to 10)
30.0 to 34.9	8 (7 to 10)
35.0 to 39.9	9 (7 to 10)
40.0 to 44.9	8 (6 to 9)
45.0 to 49.9	8 (6 to 9)
50.0 to 54.9	7 (4 to 9)
55.0 to 55.9	7 (5 to 9)
60.0 to 64.9	7 (5 to 8)
65.0 to 69.9	5 (4 to 7)
70.0 to 74.9	5 (3 to 6)
75.0 to 79.9	6 (1.5 to 6.6)
80.0 to 85.0	1 (1 to 3)

**Table 3:** CTSIB trials, showing mean and 95% confidence intervals. Use data from upper ages with caution due to small sample sizes.

Age range (years)	CTSIB Head still	CTSIB head yaw	CTSIB head pitch
4.0 to 5.9	22.9 (21.2 to 24.5)	14.9 (12.7 to 17.1)	13.8 (11.5 to 16.1)
6.0 to 7.9	25.5 (24.4 to 26.7)	18.8 (17.3 to 20.4)	16.0 (14.5 to 17.6)
8.0 to 9.9	27.0 (25.9 to 28.0)	20.1 (18.7 to 21.5)	17.7 (16.3 to 19.2)
10.0 10 11.9	27.0 (25.9 to 28.1)	21.6 (20.2 to 23.0)	17.9 (16.4 to 19.4)
12 to 13.9	28.3 (27.1 to 29.6)	22.9 (21.3 to 24.6)	17.7 (16.0 to 19.5)
14 to 15.9	26.8 (25.2 to 28.4)	22.5 (20.5 to 24.6)	19.6 (17.4 to 21.7)
16 to 17.9	27.8 (26.0 to 29.7)	26.9 (24.4 to 29.3)	22.9 (20.4 to 25.5)
18 to 19.9	26.8 (25.0 to 28.6)	23.2 (20.9 to 25.6)	21.3 (18.8 to 23.7)
20 to 24.9	28.2 (27.0 to 29.5)	24.1 (22.5 to 25.7)	22.8 (21.1 to 24.5)
25.0 to 29.9	27.6 (26.5 to 28.6)	25.5 (24.2 to 26.9)	22.3 (20.9 to 23.8)
30.0 to 34.9	28.3 (27.0 (29.6)	23.8 (22.1 to 25.5)	22.0 (20.2 to 23.8)
35.0 to 39.9	27.9 (26.5 to 29.4)	23.1 (21.2 to 24.9)	19.9 (17.9 to 21.8)
40.0 to 44.9	26.6 (25.1 to 28.0)	21.6 (19.8 to 23.5)	19.2 (17.2 (21.2)
45.0 to 49.9	24.2 (22.4 to 25.9)	18.4 (16.2 to 20.6)	16.6 (14.2 to 18.9)
50.0 to 54.9	25.3 (23.5 to 27.2)	19.4 (17.0 to 21.7)	18.5 (16.0 to 21.0)
55.0 to 55.9	22.6 (20.5 to 24.6)	19.3 (16.7 to 22.0)	18.8 (16.0 to 21.7)
60.0 to 64.9	20.6 (18.1 to 23.0)	17.0 (13.8 to 20.2)	12.4 (9.0 to 15.7)
65.0 to 69.9	20.5 (17.7 to 23.3)	15.7 (12.1 to 19.4)	12.5 (8.7to 16.4)
70.0 to 74.9	18.9 (14.9 to 23.0)	12.0 (6.8 to 17.2)	11.1 (5.6 to 16.6)
75.0 to 79.9	15.5 (10.3 to 20.6)	4.3 (0 to 11.0)	6.6 (0 to 13.7)
80.0 to 85.0	8.9 (2.4 to 15.5)	10.9 (2.5 to 19.3)	(0 to 11.5)

Males and females did not differ significantly on TW responses (p = 0.51), as shown in Table 4, with ages collapsed. They did not differ significantly on CTSIB trials with yaw (P = 0.55) and pitch head movements (p = 0.26), either, but with age groups collapsed males performed the CTSIB head still trial for significantly longer than females, p = 0.04.

**Table 4:** TW and CTSIB by sex. For TW the measure of central tendency is median (with interquartile range (I-Q)), because the number of steps is an integer. For CTSIB the measure of central tendency is mean (with standard deviation (SD)). * indicates significantly longer responses in males than females.

Test	Sex	Median (I-Q range) or Mean (SD)
TW	Female	8 (6 to 9)
	Male	8 (6 to 9)
CTSIB head still	Female	24.7 (9.3) *
	Male	25.9 (8.3) *
CTSIB head yaw	Female	20.4 (10.7)
	Male	20.8 (10.3)
CTSIB head pitch	Female	17.9 (10.7)
	Male	(10.7)

BMI was available for 1776 subjects. Those data were tested by three subgroups with sex and age groups collapsed: normal (n = 946), overweight (n = 415), and obese (n = 415). As shown in Table 5, on TW normal-weight subjects did not differ from overweight subjects, but normal and obese subjects differed significantly, p< 0.001. Overweight and obese subjects also differed significantly, p = 0.0002. Both normal and overweight groups performed significantly more correct steps than obese subjects.

**Table 5:** BMI by test and measure of central tendency. Median (I-Q range) for TW and mean (SD) for CTSIB. * and ^ indicate significant pairwise differences per test.

Test	BMI group	Median or Mean
TW	Normal	8 (7 to 10) *
	Overweight	8 (6 to 9) ^
	Obese	7 (5 to 9) *^
CTSIB head still	Normal	27.0 (7.3) *
	Overweight	25.7 (8.3) *
	Obese	25.9 (8.0)
CTSIB head yaw	Normal	21.8 (9.9)
	Overweight	21.4 (10.3)
	Obese	21.1 (10.0)
CTSIB head pitch	Normal	18.9 (10.6)
	Overweight	18.9 (10.4)
	Obese	18.6 (10.6)

On CTSIB head still, normal subjects performed the test for significantly longer than overweight, p = 0.0008. No other differences were found. The weight groups did not differ significantly on the CTSIB head yaw and head pitch conditions.

#### Educational results

The education department at Space Center Houston is tasked with informing the public about space-related research, and with educating visitors about how science is done. This study contributed to their mission by educating people about the process of science including giving Informed Consent and procedures for objective data collection. Most participants had never been research subjects before. Therefore, we asked subjects if participating in the study increased their understanding or appreciation for research. The subjects who responded Yes (N = 1626) were significantly older (mean age 25.9) than the subjects who said No (N = 208, mean age 17.4), p<0.0001. No differences were found between males and females, p = 0.27. Staff at Space Center Houston who administered the tests later informed us that most subjects who responded “No” felt that they already had a good understanding or appreciation for research. The staff reported that some children were too young to understand, but they had fun.

We asked the same subjects if, having had this research experience, they would be interested in participating in research in the future. Most subjects, n = 1833, responded. The majority (n = 1728, 94%), said they would be interested, and 6% (N-105) said they would not. The participants who were interested were significantly older than the non-interested group, (mean age 25.2 vs 20.4 yrs), p< 0.0001. No differences were found between males and females, p = 0.52.

Instructors from the Education staff who obtained informed consent, tested subjects and recorded data were subsequently asked about family participation. They stated that parents enjoyed having the entire family participate and some families even made it a game to compete with each other. They reported that if the children had fun participating then the parents would agree to participate, even parents who were initially reluctant.

We asked the staff instructors how they felt about their involvement with obtaining informed consent, testing subjects, and recording data. All instructors reported minimal to no difficulty obtaining written informed consent. One instructor reported minimal difficulty administering the tests to adults but the others reported no difficulty and seven instructors reported minor difficulty administering the tests to children. Therefore, the staff understood the test instructions and could administer the tests in this community setting. All of the staff reported that they enjoyed participating in the activity; 13 of 15 instructors said that they would like to help with another study. Staff instructors all reported that they learned something from their participation, such as information about age-related changes in balance, which was the scientific goal of the study, and also about how to educate visitors about the process of scientific research.

## Discussion

### Scientific findings

We have known for a long time that vestibularly-mediated balance scores show age-related changes [23]. This study extends that knowledge to younger children and supports the idea that vestibularly-mediated balance responses become adult-like during early [24] or late adolescence [25], depending on the study. We show additional data about decrements in balance responses later in life, supporting previous findings [11]. Different cuts to the age range might have provided different answers, so we preferred to make fairly small cuts to the age range so that the reader can decide where meaningful changes occurred. If more data were to be added to the sample, some apparent differences might disappear. For that reason, the look-up tables use small cuts to the age range, which may be more useful for clinicians than measures of central tendency for just three or four groups. Users should be cautious about the data in the four oldest age ranges because, although we are certain that the scores do decrease with age, the small samples in this study may not accurately reflect population values.

We used medium density, continuously compliant memory foam—a specific type of memory foam pad with specific performance characteristics. Many clinicians use different types of pads. For the data in Tables 2 and 3 to be useful, clinicians should administer the CTSIB using foam pads with the same performance characteristics as in this study. This problem is not trivial. Previous studies have shown that using different foam pads affects the outcome of testing [26, 27].

The data about body mass index confirm and extend previous evidence about the influence of weight on balance [16, 17, 19]. Obese individuals had poorer performance on tandem walking than non-obese individuals. Clinicians who use this test should consider BMI when comparing performance to normative data. The influence on CTSIB is less clear. The lack of differences on the CTSIB yaw and pitch conditions may be due to the greater motor challenges in those conditions, which could have outweighed the relatively slight effect of BMI.

The reason for the finding of minor sex differences on the CTSIB head still condition is not clear. This phenomenon did not occur in the head yaw and head pitch conditions. That finding may reflect greater lower extremity strength in men, as subjects learned to cope with the novel environment of the continuously compliant foam. We have previously reported a sex difference on medium firm foam, which is firmer than the foam used in this study [27]. This difference may also reflect differences in strength between males and females.

### Educational findings

Similar to previous balance testing in a different science museum (20), we were able to collect valid and useful scientific data. Unlike the previous study our education staff were directly involved to obtain informed consent and collect data and to educate the public about participating as research subjects. Most subjects had no previous experience participating in research studies, and most of them subsequently reported having had a good experience. Therefore, they were willing to consider participating in research in the future. Even young children had fun, so their parents probably considered it as a positive experience for them. Having had this good experience increases the likelihood that, when invited to participate in research in the future, these now-experienced research participants might agree.

The participants were interested in the study and appreciated the opportunity to actively contribute and engage in an authentic research experience, especially an experience that was associated with space-related research. They were excited by the idea that this activity was not just an educational exercise but, instead, they were contributing to the discovery of new knowledge. The staff instructors were able to explain how these data might be used in the future as comparison data for crew members who would be screened upon returning from exposure to microgravity, and for children and older adults. At the very least, having had a good experience participating in the activity of doing science outside of a formal classroom situation, these children and adults may be less intimidated by science and might understand the need to support scientific research in the future.

The instructors all reported having a good experience. Therefore, education staff can be tasked to help with subject recruitment and data collection without affecting their job performance. As the content of the study was directly related to current issues in human space exploration, the staff gained an invaluable experience and understanding which further enhanced education programming. The opportunity to be trained on and participate in an authentic study enabled the staff to educate the public on the need for and contribution of human health research to space exploration and human health care.

### Limitations of the study

The sample sizes in the older age ranges, especially above age 65, were small. Therefore, even though the data show the expected decrements across older ages, the size of the sample makes the actual values of the data unreliable. Had we collected a larger sample of seniors the data in Tables 1 and 2 in the upper age groups would be more likely to reflect the population. Doing so, however, was not possible in this situation. We used the population that was available at Space Center Houston, which includes children and primarily younger and middle-aged adults. Therefore, the previously reported data for older adults are probably more reliable [11].

The background noise and lack of complete privacy did not duplicate the test conditions in earlier studies, but these differences in the test environments may have been useful, inadvertently. They helped to simulate the environments in busy rehabilitation clinics and physical education programs where patients and students, respectively, do not have complete privacy and where background noise is unavoidable.

We were unable to control for socioeconomic status. That issue, however, was probably irrelevant. Space Center Houston has a strong policy of inclusivity. The Center has programs for children who are from low income school districts, a day for home-schooled children, and programs to make the Center accessible for adults and children with special needs including some health problems and mental health problems–who would not have qualified for participation in this study. The Center has a special program to encourage children to be interested in the STEM fields, i.e. science, technology, engineering and mathematics, including children from low income areas. Therefore, socioeconomic status did not preclude participation in the study.

## Supporting information

S1 Data (XLSX)
